# Anti-glycation, anti-hemolysis, and ORAC activities of demethylcurcumin and tetrahydroxycurcumin in vitro and reductions of oxidative stress in d-galactose-induced BALB/c mice in vivo

**DOI:** 10.1186/s40529-019-0258-x

**Published:** 2019-06-27

**Authors:** Yuh-Hwa Liu, Tai-Lin Lee, Chuan-Hsiao Han, Yi-Shan Lee, Wen-Chi Hou

**Affiliations:** 1 Division of Gastroenterology, Shin Kong Wu Ho-Su Memorial Hospital, Taipei, 111 Taiwan; 2 Department of General Medicine, School of Medicine, College of Medicine, Taipei Medical University, Taipei, 110 Taiwan; 3 School of Pharmacy, College of Pharmacy, Taipei Medical University, Taipei, 110 Taiwan; 4 Department of Health and Creative Vegetarian Science, Fo Guang University, Yilan, 262 Taiwan; 5 Graduate Institute of Pharmacognosy, Taipei Medical University, No. 250, Wu-Hsing Street, Taipei, 110 Taiwan

**Keywords:** Antioxidant, Curcuminoids, Glycation, Hemolytic, ORAC

## Abstract

**Background:**

There were few report concerning anti-glycation and antioxidant activities of the minor amounts of components in curcuminoids, demethylcurcumin and tetrahydroxycurcumin, in vitro and in vivo.

**Results:**

The bovine serum albumin/galactose of non-enzymatic glycation models, radical-induced hemolysis, and oxygen radical absorbance capacity (ORAC) were studied in vitro, and the d-galactose-induced oxidative stress in BALB/c mice and then demethylcurcumin or tetrahydroxycurcumin interventions in vivo. The parameters of oxidative stress in plasma and brain extracts were determined among animal groups with or without both curcuminoids interventions. The demethylcurcumin and tetrahydroxycurcumin exhibited anti-glycation, anti-hemolysis, and ORAC activities, and showed much better and significant difference (P < 0.05) compared to those of curcumin in vitro. In animal experiments, the intervened two curcuminoids at both concentrations showed to lower serum malondialdehyde (MDA), brain MDA levels and iNOS protein expressions, and elevate serum ORAC activities, and showed difference (P < 0.05) compared to the galactose-induced control.

**Conclusion:**

The demethylcurcumin and tetrahydroxycurcumin showed potentials in developing functional foods for antioxidant-related purposes.

## Background

Free radicals are extensively studied to link up their roles in several chronic diseases, such as metabolic syndrome disorders, cardiovascular and neurodegenerative diseases (Seifried et al. 2007; Hybertson et al. 2011). The intracellular oxygen-centered species (superoxide radical, hydroxyl radical, and hydrogen peroxide), also called the reactive oxygen species (ROS), are generated either by mitochondrial electron transport chains for energy productions, enzyme metabolisms (such as amine oxidase, and xanthine oxidase, etc.), or responses to environmental factors (such as UV radiation, chemicals, or pathogen attacks), which are scavenged or destroyed by enzymatic systems or non-enzymatic compounds in cells (Cui et al. 2012). The aging process of cells may be from the status of oxidative stress in which the ROS productions higher than ROS scavenging and DNA repair efficiencies, which is recognized as the theory of “free radical theory of aging” (Cui et al. 2012). The intakes of fruit and vegetable (rich in polyphenol and vitamin C) are positively correlated with the reductions of markers of inflammation (serum C-reactive protein) and oxidative stress (urinary F_2_-isoprostane) in 285 adolescences (Holt et al. 2009). The ROS-scavenging abilities and the anti-inflammatory activities of citrus flavonoids show neuroprotective effects in vitro (Hwang et al. 2012). Therefore, there are several reports concerning radical scavenging and antioxidant activities from natural resources, such as yam dioscorin and its synthesized peptides from in silico pepsin hydrolysis (Han et al. 2013, 2014a, b, c), the hydrolysable tannin of geraniin (Lin et al. 2008), and curcuminoids (Chen et al. 2006; Feng and Liu 2009; Zhao et al. 2011; Liu et al. 2016).

The Maillard reaction is a non-enzymatic glycation between carbonyl groups in reducing sugars and amino groups in proteins through nucleophilic attacks to form shiff’s base and Amadori products, and generate irreversibly advanced glycation end-products (AGEs) in the final stage (Zhang et al. 2009). The methylglyoxal and glyoxal, the metabolites of sugars and fatty acids, are also active to generate AGEs, such as the *N*^ε^-(carboxymethyl)lysine, have been structurally (Zhang et al. 2009). The AGEs can bind AGE receptors to increase ROS productions through activation of NADPH oxidase (Calcutt et al. 2009). In the use of hemoglobin as targeted proteins in vitro, the reaction rate of d-galactose is about 4.7-fold of that of d-glucose in non-enzymatic glycations (Burn and Higgins 1981). Therefore, the long-term injection of galactose is frequently used as a rodent model to induce oxidative stress (Song et al. 1999; Han et al. 2014b, c), and the spatial memory-dependent hippocampal functions is gradually decreasing (Shen et al. 2002). It is reported that the dysfunction in spatial learning and memory can be improved by antioxidant treatments (Socci et al. 1995).

The turmeric, from the rhizome of *Curcuma longa*, is used as a food spice or an alternative medicine. The term “curcuminoids” in turmeric preparations generally refer as curcumin, demethoxycurcumin (DMC), and bisdemethoxycurcumin (BDMC) (Aggarwal et al. 2007), and the minor amounts of components in curcuminoids are also reported (Li et al. 2011; Gokaraju et al. 2013), such as demethylcurcumin [1-(3,4-dihydroxyphenyl)-7-(4-hydroxy-3-methoxyphenyl)-1,6-heptadiene-3,5-dione], demethyldemethoxycurcumin (demethyl-DMC) [1-(3,4-dihydroxyphenyl)-7-(4-hydroxyphenyl)-1,6-heptadiene-3,5-dione], and tetrahydroxycurcumin [1,7-bis(3,4-dihydroxyphenyl)-1,6-heptadiene-3,5-dione]. The curcumin, DMC, and BDMC are major components in curcuminoids and have been reported to exhibit antioxidant activities in vitro (Chen et al. 2006; Feng and Liu 2009; Zhao et al. 2011; 24. Anand et al. 2008), however, the biological activity of minor amounts of components in curcuminoids are few reported. Recently, it is reported that pretreatments of demethylcurcumin and demethyl-DMC, but not curcumin, DMC and BDMC, at the same concentration showed to lower cytotoxicities of hydrogen peroxide-treated HaCaT keratinocytes (Liu et al. 2016). Therefore, in this study, the naturally occurring curcuminoids (curcumin, DMC, BDMC, demethylcurcumin, demethyl-DMC, and tetrahydroxycurcumin) were firstly assayed for anti-glycation in bovine serum albumin (BSA)/galactose models. The demethylcurcumin and tetrahydroxycurcumin were further used in vitro for anti-hemolysis and oxygen radical absorbance capacity (ORAC) activity in comparison with those of curcumin. For in vivo animal experiments, the d-galactose was injected subcutaneously to induce oxidative stress in BALB/c mice, and then demethylcurcumin and tetrahydroxycurcumin were orally administered by the gavage feeding concurrent with D-galactose injection to evaluate the changes of malondialdehyde (MDA) levels and inducible nitric oxide synthase (iNOS) protein expressions in mouse brain extracts.

## Methods

### Materials

**Fig. 1:**
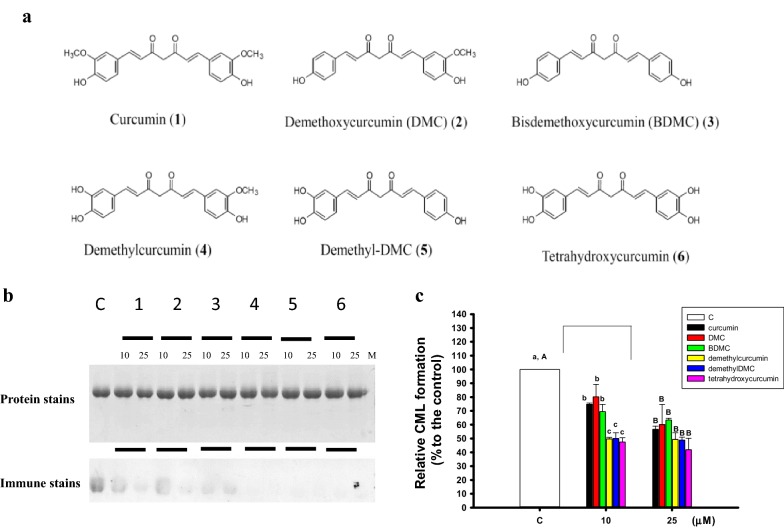
The anti-glycation activities of six curcuminoids (10 and 25 μM) in BSA/galactose models. **a** Six curcuminoids, including curcumin (**1**), demethoxycurcumin (DMC, **2**), bisdemethoxycurcumin (BDMC, **3**), demethylcurcumin (**4**), demethyl-DMC (**5**), and tetrahydroxycurcumin (**6**). **b** The protein stain on the 10% SDS-PAGE gels and the immune stain of *N*^ε^-(carboxymethyl)lysine formations on the PVDF membranes. **c** The quantification of the relative *N*^ε^-(carboxymethyl)lysine formations. Values were presented as mean ± SD and were analyzed using one-way ANOVA, followed by a post hoc Tukey’s test for multiple comparisons. The *P* < 0.05 was considered statistically significant and marked with different letters among the control and treatments under the same concentration

The curcumin (**1**), demethoxycurcumin (DMC, **2**), bisdemethoxycurcumin (BDMC, **3**), demethylcurcumin (**4**), demethyl-DMC (**5**), and tetrahydroxycurcumin (**6**) (Fig. 1a) were purchased from Laila Impex Co. Ltd. (Vijayawada, India) with purity higher than 98%. The 2,2′-azobis-(2-amidinopropane dihydrochloride) (AAPH), dimethyl sulfoxide (DMSO), d-galactose, phosphate buffered saline (PBS), and horse radish peroxidase-conjugated goat anti-rabbit IgG (A6154) were purchased from Sigma Chemical Co. (St. Louis, MO). BSA was purchased from Thermo Fisher Scientific Inc. (Rockford, IL). OxiSelect™ Assay Kit (STA-345) was from Cell Biolabs Inc. (San Diego, CA). The antibodies against iNOS and β-actin were from Santa Cruz Biotechnology (Santa Cruz, CA, USA). The anti-*N*^ε^-(carboxymethyl)lysine antibody (ab27684) was from Abcam Inc. (Cambridge, MA).

### Effects of six curcuminoids on anti-glycation in non-enzymatic BSA/galactose models detected by *N*^ε^-(carboxymethyl)lysine formations

The non-enzymatic glycation model of BSA/galactose was used to screen anti-glycation activities of six curcuminoids following the previous experiments (Han et al. 2014a; Liu et al. 2017). The total 100 μl of reaction solution contained 20 μl of BSA solution (2 mg/ml), 60 μl of 1 M galactose solution, 10 μl of PBS (10-fold dilutions), and 10 μl of six curcuminoids (the final concentrations of 10 or 25 μM in DMSO). The blank test contained BSA only, and control test contained BSA/galactose under the same conditions. These mixtures were placed at 37 °C water bath for 11 days. After being mixed with fivefold diluted sample buffer, the mixture was heated at 100 °C for 5 min, and an aliquot of 8 μl was subjected to 10% sodium dodecyl sulfate–polyacrylamide gel electrophoresis. After electrophoresis, one gel was stained with Coomassie brilliant blue G-250 solution for protein stainings, while the other gel was transferred onto immobile polyvinylidene difluoride membranes (Millipore, Bedford, MA, USA) for immune stains following the previous procedures with modifications (Liu et al. 2016). The anti-*N*^ε^-(carboxymethyl)lysine antibody was used at a 1000-fold dilution. The HRP-conjugated secondary antibody was used at a 5000-fold dilution. Immunoblots were detected by chemiluminescent system (no. WBKL S0050; Immobilon™, Millipore), and then imaged and quantified by a Syngene G:bBOX imaging system (Syngene, UK). The density in the control (BSA/galactose) was considered as 100%.

### Effects of curcumin, demethylcurcumin, and tetrahydroxycurcumin on AAPH-mediated hemolysis

The use of AAPH-mediated hemolysis of rat’s red blood cells (RBCs) in vitro was according to the previous experiments (Han et al. 2013, 2014a). After washing with 0.15 M NaCl thrice, the RBCs were harvested by centrifugation at 2000×*g* for 10 min. The curcumin, demethylcurcumin, and tetrahydroxycurcumin (5, 10, 20, and 40 μM) each was mixed with RBCs in 10 mM PBS and 50 mM AAPH solution and incubated at 37 °C for 0 to 4 h with gentle shaking. Each mixture at fixed time interval was centrifuged at 2000×*g* for 10 min, and the supernatant was collected to measure the absorbance at 536 nm. The area under the curve of absorbance at A536 nm during 4-h were calculated as the area under curve (AUC) without or with curcumin analog additions. The anti-hemolytic activities were calculated as the changes of AUC between the control and the analog addition and expressed as anti-hemolytic activity (%) by the equation of (AUC_control_ − AUC_curcumin analog_) ÷ (AUC_control_ − AUC_blank_) × 100%.

### The ORAC activity of curcumin, demethylcurcumin, and tetrahydroxycurcumin in vitro

The ORAC activity determination in vitro was according to the previous methods (Han et al. 2013, 2014a) by using OxiSelect™ Assay Kit. The antioxidant samples in the assay system can block peroxyl (such as AAPH) radical-mediated fluorescein oxidation during 60 min by determining the ratio of Ex480 nm/Em520 nm. The area under each curve of Ex480 nm/Em520 nm without or with curcuminoids or Trolox additions during 60-min were calculated as the AUC. The Trolox (2.5, 5, 10, 20, 40, and 60 μM) were used to plot standard curve between AUC and Trolox concentrations. The ORAC activity of curcumin, demethylcurcumin, and tetrahydroxycurcumin was interpolated AUC and was expressed as μM Trolox equivalents (μM TE).

### Effects of oral administrations of demethylcurcumin or tetrahydroxycurcumin on reductions of oxidative stress in galactose-induced mice

The oral administration of demethylcurcumin or tetrahydroxycurcumin were used to evaluate the improvements of oxidative stress in galactose-induced mice. The male BALB/c mice, 8-week-old (N = 36), were purchased and provided by National Laboratory Animal Center (Nangang, Taipei), and housed in a well-controlled temperature and humidity environment. These animal experimental protocols have been reviewed and approved by the Institutional Animal Care and Use Committee of Taipei Medical University (LAC-99-0142). The mice had free access to normal feeds (Prolab^®^ RMH2500, MO) and water, after two-week acclimations, mice were randomly divided into one blank and five galactose-induced groups without or with demethylcurcumin or tetrahydroxycurcumin interventions. These six groups included: (i) the blank group; (ii) the galactose-induced group for 8 weeks (the control group); (iii) 5 mg/kg demethylcurcumin intervention for 4 weeks; (iv) 10 mg/kg demethylcurcumin intervention for 4 weeks; (v) 5 mg/kg tetrahydroxycurcumin intervention for 4 weeks; and (vi) 10 mg/kg tetrahydroxycurcumin intervention for 4 weeks. The 12% galactose (in normal saline) was injected subcutaneously to the dorsal necks of BALB/c mice once a day for 8 weeks (injection volume, 0.1 ml/10 g mouse body weight). The intervention groups at doses of 5 or 10 mg/kg of body weight (group iii to group vi) were orally administered once a day by the gavage feeding in the beginning of 5th week for 4 weeks concurrent with galactose injections. For the blank group, each mouse was injected with normal saline for 8 weeks, and distilled water was administered orally in the beginning of 5th week once a day for 4 weeks.

### Effects of oral administrations of demethylcurcumin or tetrahydroxycurcumin on oxidative stress parameters in vivo

After being sacrificed, blood samples of mice were collected for determining oxidative stress parameters, and the mice brains were isolated and stored at − 80 °C for further measurements. The whole brains in liquid nitrogen were powdered using a mortar and pestle and then suspended in 1 ml of 1X PBS on the ice bath. After being centrifuged at 10,000×*g* at 0 °C for 30 min, the supernatants were collected and stored at − 80 °C for further investigation. The proteins in brain extracts were quantified by the BCA protein assay kit (Thermo Fisher Scientific Inc., USA) using BSA as the standard protein. The MDA content in plasma or in the brain extracts (10 μg protein) was determined by BIOXYTECH^®^ MDA-586™ assay kits (Portland, OR, USA), following the instructions. The ORAC activities in plasma or in the brain extracts (10 μg protein) were determined using the OxiSelect™ assay kit as above-mentioned method according to the manufacturer’s instructions. The area under the curve of Trolox was used to plot a standard curve of ORAC activity and expressed as μM TE. For iNOS protein expression, 40 μg proteins of brain extracts in each group were subjected to 10% sodium dodecyl sulfate–polyacrylamide gel electrophoresis. After electrophoresis, the gels were transferred onto PVDF membranes (Millipore, MA) and then following the previous report with modifications (Liu et al. 2016). The iNOS antibody was used in a 2000-fold dilution, and β-actin was used in a 10,000-fold dilution. The images were quantified and expressed as folds of iNOS/actin by the relative density (%) to the control (100%).

### Statistical analysis

Values are presented as mean ± SD and were analyzed using one-way ANOVA, followed by a post hoc Tukey’s test for multiple comparisons. The *P* value less than 0.05 was considered statistically significant and marked on the column with different letters among treatments or treatments under the same concentration. The statistical analysis was performed using the GraphPad Prism Software 5.0.

## Results

### Anti-glycation activities in BSA/galactose models

The non-enzymatic BSA/galactose models were used to investigate the effects of six curcuminoids (10 and 25 μM) on anti-glycation, which the protein stains of BSA and N^ε^-(carboxymethyl)lysine formations showed in Fig. 1b. There was no apparent difference of BSA protein stains in the SDS-PAGE gel among the control and six curcuminoids treatments. However, the N^ε^-(carboxymethyl)lysine intensities in the control showed the highest one, and much lower levels were found after six curcuminoids treatments (10 and 25 μM) and showed a significant difference compared to the control (*P* < 0.05), and the quantified CML intensities showed in the Fig. 1c. At 10 μM concentration, demethylcurcumin (lane 4), demethyl-DMC (lane 5), and tetrahydroxycurcumin (lane 6) showed to lower over 50% N^ε^-(carboxymethyl)lysine formations in BSA protein, and exhibited much better and showed significant differences of anti-glycation activities (*P* < 0.05) compared to those of curcumin (lane 1), DMC (lane 2), and BDMC (lane 3). There was no significant difference (*P* > 0.05) of anti-glycation activity among six curcuminoids at 25 μM.

### Anti-hemolytic and ORAC activities

**Fig. 2:**
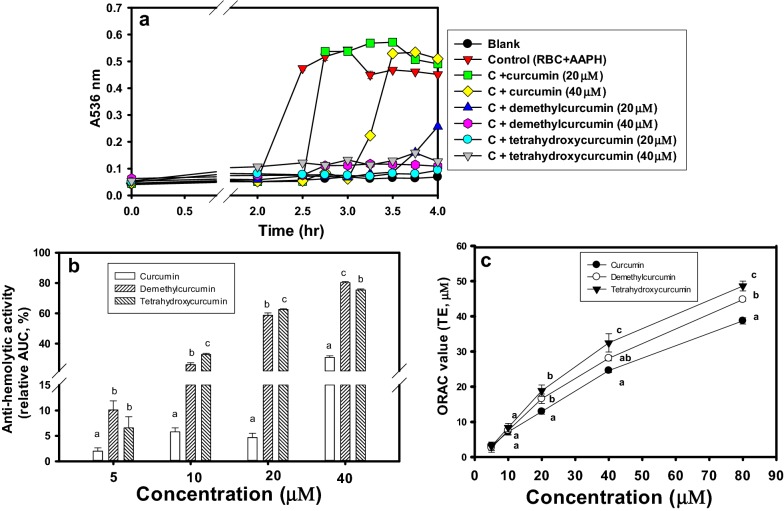
Anti-hemolytic and ORAC activities of curcumin, demethylcurcumin, and tetrahydroxycurcumin in vitro. **a** The typical profiles of three curcuminoids (20 and 40 μM) on protections against AAPH-mediated hemolysis during 4 h (expressed as A536 nm). **b** The quantification of anti-hemolysis (%) of three curcuminoids (5, 10, 20, and 40 μM) by calculating relative AUC. **c** The ORAC activities of three curcuminoids and expressed as Trolox equivalents (μM TE). Values were presented as mean ± SD and were analyzed using one-way ANOVA, followed by a post hoc Tukey’s test for multiple comparisons of anti-hemolytic activities. The *P* < 0.05 was considered statistically significant and marked with different letters among treatments under the same concentration

The demethylcurcumin and tetrahydroxycurcumin were selected in comparison with curcumin for further anti-hemolytic activities and shown in Fig. 2a, b. The AAPH radical showed to induce hemolysis (the control) by increasing A536 nm after 2-h reactions, and in the absence of AAPH (the blank), no apparent hemolysis was found. The curcumin at 20 and 40 μM showed to delay hemolysis (A536 nm) from 2 to 2.75-h and 3.25-h, respectively. The demethylcurcumin or tetrahydroxycurcumin under the same concentrations showed completely anti-hemolysis (A536 nm) during 3.5-h incubations. The quantification of anti-hemolytic activities by calculating the net AUC between the control and each treated sample showed in Fig. 2b. The curcumin, demethylcurcumin, and tetrahydroxycurcumin exhibited dose-dependent anti-hemolytic activities, which the demethylcurcumin and tetrahydroxycurcumin showed much higher anti-hemolytic activities and had a significant difference (*P* < 0.05) compared to that of curcumin at the same concentration.

The ORAC activities in vitro of curcumin, demethylcuircumin, and tetrahydroxycurcumin at concentrations of 5, 10, 20, 40, and 80 μM showed in Fig. 2c. These three curcuminoids showed dose-dependent ORAC activities, and the orders of ORAC activities under the same concentration (especial for concentrations higher than 40 μM) were tetrahydroxycurcumin > demethylcurcumin > curcumin. From the results of Fig. 2, the demethylcurcumin and tetrahydroxycurcumin showed better antioxidant activities ahgainst AAPH radical-mediated hemolysis and fluorescence-decay than those of curcumin.

### Effects of demethylcurcumin or tetrahydroxycurcumin interventions on reductions of oxidative stress in galactose-treated mice

**Fig. 3:**
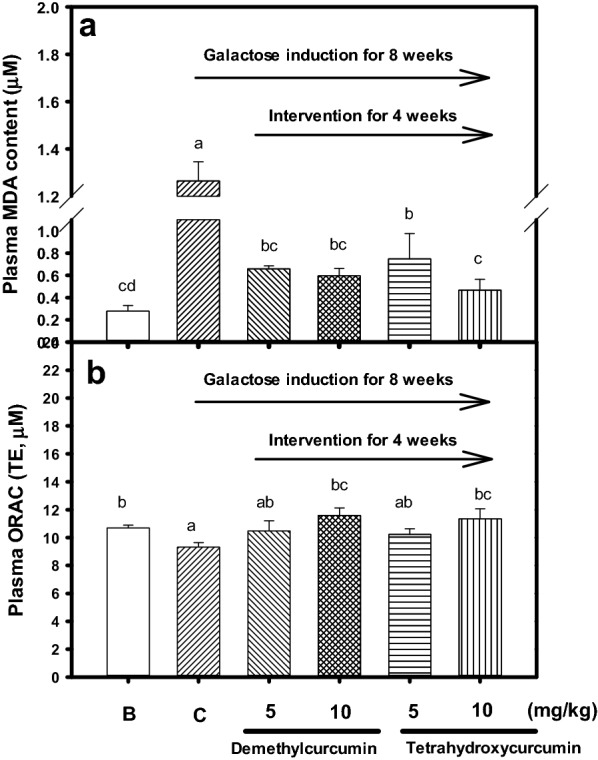
Effects of demethylcurcumin or tetrahydroxycurcumin interventions (5 and 10 mg/kg) on **a** plasma MDA contents, and **b** plasma ORAC activities in d-galactose-induced mice. Values were presented as mean ± SD and were analyzed using one-way ANOVA, followed by a post hoc Tukey’s test for multiple comparisons. The *P* < 0.05 was considered statistically significant and marked on the column with different letters among treatments

The plasma MDA contents and plasma ORAC activities of intervened mice after 4-week oral administrations of demethylcurcumin or tetrahydroxycurcumin after galactose-induced oxidative stress mice showed in Fig. 3. The plasma MDA contents of mice were elevated in the control group and showed a significant difference compared to that of the blank (*P* < 0.05). The demethylcurcumin or tetrahydroxycurcumin interventions at concentrations of 5 and 10 mg/kg concurrent with galactose injection showed to reduce plasma MDA contents of mice and had significant differences compared to those in the control (*P* < 0.05) (Fig. 3a). The plasma ORAC activities of mice were reduced in the control group and showed a significant difference compared to that of the blank (*P* < 0.05). While, the demethylcurcumin or tetrahydroxycurcumin interventions at concentrations of 5 and 10 mg/kg concurrent with galactose injection showed to elevate plasma ORAC activities of mice and had significant differences at 10 mg/kg compared to those in the control (*P* < 0.05) (Fig. 3b).

**Fig. 4:**
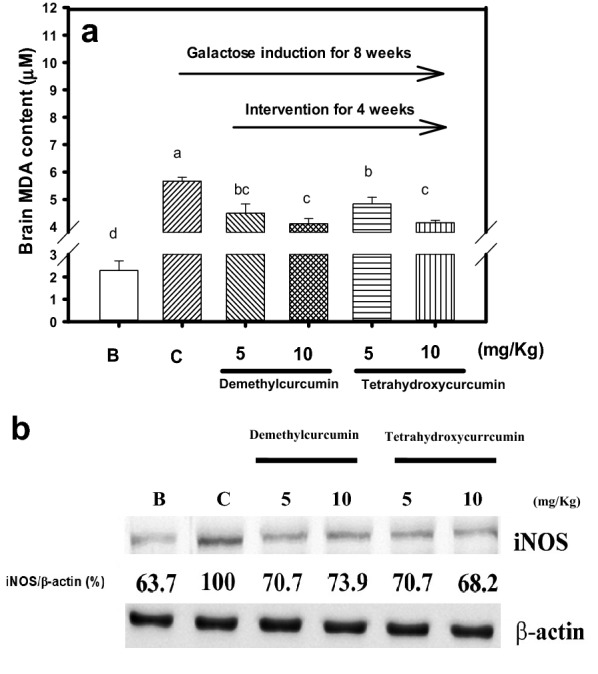
Effects of demethylcurcumin or tetrahydroxycurcumin interventions (5 and 10 mg/kg) on brain extracts of **a** MDA contents and **b** iNOS protein expressions in d-galactose-induced mice. The iNOS antibody was used in a 2000-fold dilution, and β-actin was used in a 10,000-fold dilution. The images of the immune stain on the PVDF membranes were quantified and expressed as folds of iNOS/actin by the relative density (%) to the control (100%). Values were presented as mean ± SD and were analyzed using one-way ANOVA, followed by a post hoc Tukey’s test for multiple comparisons. The *P* < 0.05 was considered statistically significant and marked on the column with different letters among treatments

The MDA levels of mice in the brain extracts were elevated in the control group, and had a significant difference (*P* < 0.05) compared to that of the blank (Fig. 4a). While, the demethylcurcumin or tetrahydroxycurcumin interventions at concentrations of 5 and 10 mg/kg concurrent with galactose injection showed to lower MDA contents in brain extracts of mice and had significant differences compared to those in the control (*P* < 0.05) (Fig. 4a). It was found that demethylcurcumin or tetrahydroxycurcumin interventions showed to lower iNOS protein expressions in brain extracts of mice compared to that of the control from 100% to about 70% (Fig. 4b).

## Discussion

The natural curcuminoids of three minor components in curcuminoids, demethylcurcumin, demethyl-DMC, and tetrahydroxycurcumin, showed better anti-glycation activities compared to those of major components in curcuminoids (curcumin, DMC, and BDMC) at 10 μM low concentrations (Fig. 1). The demethylcurcumin, demethyl-DMC, and tetrahydroxycurcumin showed better anti-glycation activities by lowering N^ε^-(carboxymethyl)lysine formations than those of major components in curcuminoids. The AGEs can bind AGE receptor to increase ROS productions through activation of NADPH oxidase (Calcutt et al. 2009), and also reported to correlate with cardiovascular disease and diabetes complications (Thornalley 2003; Yamagishi et al. 2008). The natural flavonoids (Wu and Yen 2005), *S*-allyl cysteine and aged gallic extracts (Ahmad et al. 2007), clinical drugs of acetohydroxamic acid, hydroxyurea, and aminoguanidine (Liu et al. 2017), were reported to reduce AGEs formations. The mechanisms of anti-glycation for curcuminoids were not clear. However, it was reported that the bovine liver catalase lost its activities during non-enzymatic glycation reaction, and curcumin additions showed to recover the catalase activity during glycation reactions (Najjar et al. 2017), which was proposed that curcumin might play roles in the ROS scavenging, the decreases of the accessible surface area of protein itself, and the increases the pKa of Lys residue of catalase for anti-glycation activity. It is possible that the anti-glycation activities of demethylcurcumin, demethyl-DMC, and tetrahydroxycurcumin might exhibit similar mechanisms to above-mentioned report (Najjar et al. 2017), which need further investigations.

Considering the point of the chemical structure among curcuminoids used in the present study, both of demethylcurcumin and demethyl-DMC contained three hydroxyl groups, and tetrahydroxycurcumin owned four hydroxyl groups. The major components of curcuminoids, curcumin, DMC, and BDMC, all contained two hydroxyl groups (Fig. 1a). It was reported that the phenolic OH groups in curcuminoids were important for their the antioxidant activities, and the more hydroxyl groups in structures, the higher antioxidant capacities were found in vitro (Feng and Liu 2009). Therefore, demethylcurcumin and tetrahydroxycurcumin were selected in comparison with curcumin for increasing anti-hemolysis and ORAC activities in vitro. The delaying AAPH-induced hemolysis and AAPH-induced fluorescent decays of ORAC activities were in the order of tetrahydroxycurcumin > demethylcurcumin > curcumin (Fig. 2), which were generally matched with the numbers of hydroxyl groups in each molecules. It was reported that anti-lipid peroxidation of three major components in curcuminoids were in the order of curcumin > DMC > BDMC in the linoleic acid models, the erythrocyte membrane systems, and the rat liver microsomal systems determined by the thiobarbituric acid method (Osawa et al. 1995). The curcuminoids in turmeric preparations contained 77% curcumin, 17% to 18% DMC, and 5% to 6% BDMC in general (Aggarwal et al. 2007; Anand et al. 2007). The tetrahydroxycurcumin was minor components about 0.1 to 5% in the natural curcumin extracts, which might be enriched to 50% to 100% by Lewis acid catalyst-aided demethylation processes and chromatographic techniques, and demethylcurcumin could also be isolated by column chromatography in enriched tetrahydroxycurcumin fractions (Gokaraju et al. 2013). The biotransformation method was reported to enrich demethylcurcumin or tetrahydroxycurcumin from curcumin, in which curcumin was reported to biotransform to generate demethylcurcumin, and in advance to tetrahydroxycurcumin by human intestinal bacteria, *Blautis* sp. MRG-PMF1 (Burapan et al. 2017). Therefore, the bioactive and minor components in curcuminoids, demethylcurcumin and tetrahydroxycurcumin, might be available in the future.

Curcumin, the most abundant curcuminoids in nature, has been intensively studied and reported the low bioavailability property by the poor water solubility, low absorption, and rapid metabolism (Anand et al. 2007; Prasad et al. 2014). The present animal models showed that the daily oral administration of demethylcurcumin or tetrahydroxycurcumin at 5 and 10 mg/kg could directly or indirectly regulate oxidative stress in galactose-induced mice. The long-term galactose injection was one aging model in rodents, which the ROS and AGEs generations might destroy spatial memories (Song et al. 1999; Kumar et al. 2011). It was reported that galactose-induced mice with impairment in spatial learning and memory capacities could be improved by 15 and 30 mg/kg curcumin treatments by Morris water maze (Kumar et al. 2011). From the results of animal experiments, demethylcurcumin or tetrahydroxycurcumin interventions was shown to improve the oxidative stress parameters compared to those in the control, including the reduced MDA levels in plasma (Fig. 3a) and brain extracts (Fig. 4a) of mice, and the elevated plasma ORAC activities (Fig. 3b) of mice. AGEs can interact with the receptor for AGE to promote ROS production or via NF-κB signaling pathways to express proinflammatory cytokines, such as TNF-α and IL-6 (Calcutt et al. 2009). The continuous inflammations in neuron cells can induce cell damages and gradually develop the neurodegenerative disorders, such as Alzheimer’s disease and Parkinson’s disease (Li et al. 2016). The iNOS protein expressions (Fig. 4b) in brain extracts of intervened mice reduced and had significant difference compared to those in the control, which meant that the inflammatory mediators were reduced after demethylcurcumin or tetrahydroxycurcumin interventions. It has been reported that demethylcurcumin, demethyl-DMC, and tetrahydroxycurcumin, but not curcumin, showed to increase neprilysin activity in cell-based models, which was associated with Aβ degradations; and the demethylcurcumin intervention could elevate neprilysin activity and lower Aβ accumulation in the hippocampus and cortex of the transgenic APP_swe_/PS_1_dE_9_ mice (Chen et al. 2016). It was possible that the demethylcurcumin or tetrahydroxycurcumin interventions at dose of 5 or 10 mg/kg not only reduce oxidative stress in galactose-induced mice but also can attenuate the impairment in spatial learning and memory which will investigate further.

## Conclusion

In conclusion, the minor components in curcuminoids of demethylcurcumin and/or tetrahydroxycurcumin showed anti-glycation, anti-radical-induced hemolysis, and antioxidant activities better than those of curcumin in vitro, and also exhibited improvements against oxidative stress induced by chronic galactose-injected mice in vivo. It is possible to enrich the minor components in curcuminoids to develop functional foods for antioxidant-related purposes.

## Data Availability

All data generated during the study are interpreted in the manuscript. The curcuminoids samples used in the present research are available in the lab of corresponding author, which were purchased from Laila Impex Co. Ltd. (Vijayawada, India).
