# Correction to: Anti-glycation, anti-hemolysis, and ORAC activities of demethylcurcumin and tetrahydroxycurcumin in vitro and reductions of oxidative stress in d-galactose-induced BALB/c mice in vivo

**DOI:** 10.1186/s40529-020-00285-3

**Published:** 2020-03-09

**Authors:** Yuh-Hwa Liu, Tai-Lin Lee, Chuan-Hsiao Han, Yi-Shan Lee, Wen-Chi Hou

**Affiliations:** 1 Division of Gastroenterology, Shin Kong Wu Ho-Su Memorial Hospital, Taipei, 111 Taiwan; 2 Department of General Medicine, School of Medicine, College of Medicine, Taipei Medical University, Taipei, 110 Taiwan; 3 School of Pharmacy, College of Pharmacy, Taipei Medical University, Taipei, 110 Taiwan; 4 Department of Health and Creative Vegetarian Science, Fo Guang University, Yilan, 262 Taiwan; 5 Graduate Institute of Pharmacognosy, Taipei Medical University, No. 250, Wu-Hsing Street, Taipei, 110 Taiwan

## Correction to: Bot Stud (2019) 60:9 10.1186/s40529-019-0258-x

In the publication of this article (Liu et al. 2019), there was an error in the method and ethics declarations sections which were published with incorrect animal experiment approval number. The error: ‘These animal experimental protocols have been reviewed and approved by the Institutional Animal Care and Use Committee of Taipei Medical University (LAC-99-0142).’ Should instead read: These animal experimental protocols have been reviewed and approved by the Institutional Animal Care and Use Committee of Taipei Medical University (LAC-2016-0340).
